# A bonus task boosts people's willingness to offload cognition to an algorithm

**DOI:** 10.1186/s41235-024-00550-0

**Published:** 2024-04-23

**Authors:** Basil Wahn, Laura Schmitz

**Affiliations:** 1 Institute of Educational Research, Ruhr University Bochum, Bochum, Germany; 2 Department of Cognitive Psychology and Ergonomics, Technische Universität Berlin, Berlin, Germany; 3 Department of Neurology, University Medical Center Hamburg-Eppendorf, Hamburg, Germany

**Keywords:** Cognitive offloading, Human–computer collaboration, Human–computer interaction, Social cognition, Algorithmic aversion, Algorithmic appreciation

## Abstract

With the increased sophistication of technology, humans have the possibility to offload a variety of tasks to algorithms. Here, we investigated whether the extent to which people are willing to offload an attentionally demanding task to an algorithm is modulated by the availability of a bonus task and by the knowledge about the algorithm’s capacity. Participants performed a multiple object tracking (MOT) task which required them to visually track targets on a screen. Participants could offload an unlimited number of targets to a “computer partner”. If participants decided to offload the entire task to the computer, they could instead perform a bonus task which resulted in additional financial gain—however, this gain was conditional on a high performance accuracy in the MOT task. Thus, participants should only offload the entire task if they trusted the computer to perform accurately. We found that participants were significantly more willing to completely offload the task if they were informed beforehand that the computer’s accuracy was flawless (Experiment 1 vs. 2). Participants’ offloading behavior was not significantly affected by whether the bonus task was incentivized or not (Experiment 2 vs. 3). These results combined with those from our previous study (Wahn et al. in PLoS ONE 18:e0286102, 2023), which did not include a bonus task but was identical otherwise, show that the human willingness to offload an attentionally demanding task to an algorithm is considerably boosted by the availability of a bonus task—even if not incentivized—and by the knowledge about the algorithm’s capacity.

**Supplementary Information:**

The online version contains supplementary material available at 10.1186/s41235-024-00550-0.

## Significance statement

In today’s world, complex cognitive tasks formerly reserved for humans start becoming feasible for computer algorithms. Consequently, humans encounter increasingly more opportunities to offload a variety of tasks to such algorithms. The present study investigates under which conditions people engage in this form of “cognitive offloading”. Our findings demonstrate that people’s willingness to offload an attentionally demanding task to a computer algorithm is influenced by their knowledge about the algorithm’s capacity and by the possibility to engage in a bonus task. That is, people want to make sure that the offloaded work is performed well: they are more willing to offload if they have knowledge about the algorithm’s reliability. Also, people want to avoid boredom and stay engaged: they are more willing to offload if they themselves have another task to perform—regardless of whether this task promises additional monetary reward.

## Introduction

In today’s world, algorithms have been seamlessly integrated into the daily life of many people. Indeed, for a variety of tasks, we almost naturally rely on algorithms—be it when planning a route with the help of *Google Maps* (or a similar navigation app), when using a search engine on the internet, or when letting a thermostat control the heating in our home. Even complex cognitive tasks that used to be reserved for humans start becoming feasible for algorithmic artificial systems, such as driving a car (via automated driving systems) or writing a poem (via chatbots such as *ChatGPT*). Note that in the present article, we use the term “artificial systems” to refer to basic algorithmic systems *without* learning capabilities. We do *not* refer to—and thus do not aim to make claims about—more advanced AI technology that involves machine learning (such as self-driving cars and generative chatbots).

Despite the apparent prevalence of artificial systems in daily life, humans show diverging attitudes when it comes to trusting an algorithm, instead of another human, to perform certain tasks (Bigman & Gray, 2018; Dietvorst et al., 2015; Logg et al., 2019). Studies comparing the human willingness to offload tasks to another human vs. an artificial system have found so-called algorithmic aversion (i.e., a preference for humans over algorithms; see Bigman & Gray, 2018) as well as algorithmic appreciation (i.e., a preference for algorithms over humans; see Logg et al., 2019). Whether people tend to show aversion or appreciation toward algorithms depends on a multitude of factors (for a recent review, see Jussupow et al., 2020). One such factor is the extent of autonomy an algorithm possesses. In particular, people tend to show aversion if an algorithm performs an entire task autonomously (Dietvorst et al., 2015). This tendency for aversion is observed especially once the algorithm commits an error—even if, overall, the algorithmic performance is still more accurate than that of a human (Dietvorst et al., 2015). In contrast, if an algorithm is not completely autonomous but takes on an advisory role only, humans tend to show appreciation (Logg et al., 2019), especially if the algorithm is known to be more capable than a human (Bigman & Gray, 2018). Thus, overall, people seem more likely to feel aversion toward an algorithm that performs a task autonomously compared to an algorithm in an advisory role.

Whereas the above-mentioned studies on algorithmic aversion/appreciation directly compared the human willingness to offload tasks to another human vs. an artificial system, other studies have focused on the conditions under which humans are generally willing to offload cognition to artificial systems (Wahn et al., 2023; Weis & Wiese, 2019a, 2019b, 2022; Wiese et al., 2022). Such cognitive offloading, broadly defined as “the use of physical action to alter the information processing requirements of a task so as to reduce cognitive demand” (Risko & Gilbert, 2016, p. 676), can help humans to overcome cognitive capacity limitations (Marois & Ivanoff, 2005; Wahn & König, 2017) and thereby enable them to attain goals they could not have attained (as quickly, easily, or efficiently) otherwise. Cognitive offloading is omnipresent in daily life. For example, by using a navigation app such as *Google Maps* to navigate in a foreign city, people reduce their own cognitive effort and free up capacity for other tasks. More recently, people have started offloading even highly complex (and creative) cognitive tasks, e.g., when using chatbots such as *ChatGPT* to generate entire text documents (e.g., poems, essays, or paper abstracts), thereby minimizing the cognitive effort they exert themselves. Given that, in the near future, humans will encounter increasingly more such opportunities to offload cognition to artificial systems, it is timely to further investigate the factors influencing whether and to what extent humans are willing to engage in such offloading.

Previous research has already investigated cognitive offloading in a wide range of contexts (see Risko and Gilbert (2016), for a review) and has identified a number of tasks and factors that influence the human willingness to offload cognition (Weis & Wiese, 2019a, 2019b, 2022; Wiese et al., 2022). In particular, one such factor seems to be the capability humans ascribe to an artificial system. This was suggested, for instance, by Weis and Wiese (2019a, 2019b) who asked participants to solve a mental rotation task and gave them the option to manipulate a knob to physically rotate the given stimulus. Thus, participants could reduce their own cognitive effort by offloading the rotation task to the knob. Importantly, the (actual and believed) reliability of that knob was systematically varied. Results showed that participants used the knob less frequently if its reliability was (believed to be) lower. In another study (Weis & Wiese, 2022), participants were required to solve an arithmetic or a social task with the aid of an “assistant” which could be either another human, a robot, or an app. These assistants were described as having either task-specific or task-unspecific expertise. It was found that offloading behavior was greatly influenced by the different descriptions (i.e., more offloading for task-specific vs. task-unspecific expertise), yet only for the app and less so for the human and robot. Moreover, it was found that participants preferred to offload the arithmetic task to robots that were described as being specifically skilled only in the arithmetic domain (Wiese et al., 2022). Together, these previous findings suggest that people's willingness to offload tasks to artificial systems can be influenced by the system’s (actual and believed) reliability (Weis & Wiese, 2019a, 2019b) and by its (ascribed) expertise (Weis & Wiese, 2022; Wiese et al., 2022).

### Previous study

In our own recent study (Wahn et al., 2023), we extended the line of research on cognitive offloading by investigating offloading in a context with high attentional demands. Specifically, we asked whether and to what extent humans offload parts of a spatial attention task to an algorithm—and whether prior information about the capability of this algorithm affects the human tendency for offloading. To address this question, we conducted two behavioral experiments in which participants performed a multiple object tracking (MOT) task (Pylyshyn & Storm, 1988). This task was chosen because it is frequently used to investigate the limits of attentional processing (Alvarez & Franconeri, 2007). Moreover, it has been previously used in studies on human–human (Wahn et al., 2021a) and human–computer collaboration (Wahn & Kingstone, 2021; Wahn et al., 2021b). In the MOT task, participants are asked to track a subset of moving target objects among distractor objects on a computer screen. The human tracking capacity is limited such that participants can typically track maximally four objects (Intriligator & Cavanagh, 2001; but also see: Alvarez & Franconeri, 2007). It has also been shown that tracking is effortful and requires sustained attention over prolonged periods of time (e.g., Alnæs et al., 2014; Wahn et al., 2016). In our previous study (Wahn et al., 2023), participants first performed the MOT task alone (Solo condition) and then had the opportunity to offload an unlimited number of targets to a computer partner (Joint condition). Based on earlier research, we had two opposing hypotheses: On the one hand, we predicted that participants might not offload the MOT task to an autonomous algorithm because humans generally seem to prefer algorithms that have an advisory function only (Dietvorst et al., 2015; see above). On the other hand, given that the MOT task is attentionally demanding, we predicted that participants might (partially) offload the task to an algorithm to reduce their own cognitive effort (Risko & Gilbert, 2016)—in line with the offloading tendencies humans show in daily activities. Furthermore, we reasoned that the second hypothesis seemed more likely because we used an experimental scenario with rather low stakes and human offloading tendencies may arguably be stronger when no high stakes are involved (see Wahn et al., 2023).

Indeed, the results of our previous study showed that participants significantly offloaded some (but not all) targets to the computer partner, thereby improving their individual tracking accuracy. Specifically, participants tracked three targets on average in the Solo and two targets in the Joint condition, i.e., they offloaded one target to the computer partner. In a second experiment, participants were informed beforehand that the computer partner’s tracking accuracy was flawless (in fact though, the computer performed at 100% accuracy in both experiments). The results of the second experiment showed a similar tendency for offloading as observed in the first experiment.

In sum, our previous study (Wahn et al., 2023) indicates that humans are willing to offload parts of an attentionally demanding task to an algorithm. An open question that remained was the following: Why did participants not offload the *complete* task to the computer partner—especially in the second experiment when they knew that the computer would perform the task flawlessly? In our previous work, we suggested two potential explanations. One explanation is that participants did not want to feel bored while passively waiting for the computer to finish the task. In other words, they preferred actively tracking some of the targets in order to stay engaged, rather than handing over all targets to the computer and not having any task of their own. Support for this hypothesis comes from a recent study which showed that people sometimes actually prefer cognitive effort over doing nothing (Wu et al., 2023). An alternative explanation is that participants experienced some form of experimenter demand, i.e., they wanted to continue performing the task that the experimenter had asked (and paid) them to perform.

### Present study

In the present study, we aimed to understand whether the aforementioned reasons might have prevented participants in our previous study from offloading the entire task to the computer partner. To this end, we adjusted the task in a way that should facilitate participants’ willingness to offload. In particular, we informed participants in the present study that, if they were to offload the MOT task entirely to the computer partner, then they could perform a bonus task while the computer performed the MOT task. This way, participants should feel free to offload the entire task—they should not feel bored nor should they have the impression to fall short of the experimenter’s demands. Moreover, we reasoned that giving participants the chance to perform a bonus task would arguably create a higher resemblance with offloading situations in daily life when people decide to offload a certain task to an artificial system (e.g., let *Google Maps* compute the fastest route) in order to free up capacity to perform another task themselves (e.g., start the car). In a second step, we tested whether participants would perform the bonus task only if it was financially incentivized or also if it came without additional incentive.

As in our previous study and in line with earlier research (see above; Weis & Wiese, 2019a, 2019b, 2022; Wiese et al., 2022), we included the computer partner’s capacity as an additional factor in the present study. That is, we varied whether participants were informed about the computer’s capacity or not, such that participants either did not receive any information about the computer’s capacity (Experiment 1) or were explicitly informed that the computer was flawless (Experiment 2). As in our previous study, the computer’s tracking accuracy was actually 100% in both experiments. Experiment 3 was identical to Experiment 2 except that the bonus task was not incentivized (see below for details).

## Materials and methods

### Participants

We collected data from 78 university students, with 26 participants taking part in each of our three experiments. All students attended the same university (and most of them were enrolled in the same study program) and the three participant samples were comparable in age range and gender identity ratio, with Experiment 1 (*M* = 25.96 years, SD = 5.67 years, 19 female, 6 male, 1 diverse), Experiment 2 (*M* = 25.58 years, SD = 5.33, 22 female, 4 male), and Experiment 3 (*M* = 23.69 years, SD = 3.97 years, 20 female, 5 male, 1 diverse). The sample size was matched to our previous study (Wahn et al., 2023).^1^ Additionally, a Power analysis conducted with G*Power (Faul et al., 2009) showed that the chosen sample size provides sufficient power to detect medium-sized effects for within-subjects comparisons (Cohen’s *d* = 0.58, Power = 0.80; alpha = 0.05, two-sided paired samples *t*-test) and large effects for between-subjects comparisons (Cohen’s *d* = 0.80, Power = 0.80; alpha = 0.05, two-sided two-sample *t*-test). Participants gave their informed consent prior to the experiment and received 15 EUR as compensation (base payment). If they chose to perform the bonus task, they could additionally earn up to 5 EUR or lose 5 EUR off the base payment (only in Experiments 1 and 2; for details, see below). The Ethics Committee of the Institute of Philosophy and Educational Research at Ruhr University Bochum approved the study (EPE-2023–003).

### Experimental setup and procedure

We followed the same procedure as in our previous study (Wahn et al., 2023). The only difference was that we integrated the above-mentioned bonus task into our experimental design.

Participants were seated at a distance of 90 cm in front of a 24′′ computer screen (refresh rate: 60 Hz, resolution: 1920 × 1080), with a keyboard and mouse placed within easy reach. Experiment 1 started with a Solo condition in which participants performed the MOT task alone. This was followed by a Joint condition in which participants were given the option to offload (parts of) the MOT task to a so-called computer partner (i.e., an algorithm). The two conditions were always performed in this order because we wanted participants to first learn about their own maximum tracking load (i.e., the number of targets they could successfully track) before deciding how many targets to offload to the computer. Before participants started the Solo condition, the experimenter explained the task procedure and instructed participants to perform two training trials so that they could familiarize themselves with the procedure. Another two training trials were performed before the start of the Joint condition. The experiment consisted of 75 trials in total, with 25 trials in the Solo and 50 trials in the Joint condition.

#### Solo condition

**Fig. 1:**
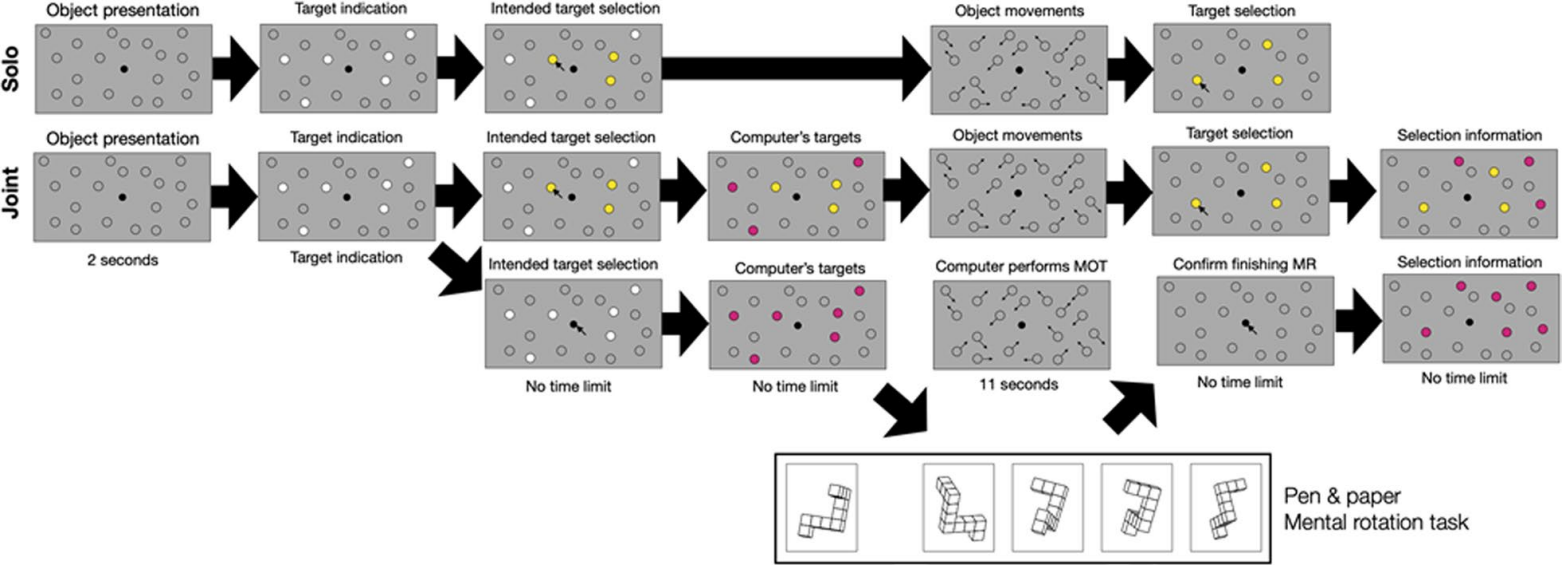
1st row: Example trial for the Solo condition. 2nd and 3rd row: Example trial for the Joint condition when participants chose to offload a subset of targets to the computer partner (2nd row) or to offload all targets (i.e., the entire MOT task) to the computer partner and to perform the mental rotation (MR) task instead (3rd row)

Each trial started with a display of 19 stationary objects (circles) on the screen (see Fig. 1). Out of these, six randomly selected objects were highlighted in white—these were the “targets”. The remaining 13 objects served as “distractors” and were colored in gray. Participants were instructed to select all those targets (i.e., between 0 and 6) that they wanted to track in that trial. To do so, participants selected the targets via mouse click and then confirmed the selection by clicking on a dot in the center of the screen. Once the selection was confirmed, the highlighted targets switched color such that all objects (targets and distractors) were now colored in gray and looked indistinguishable. All objects then started moving across the screen in randomly selected directions. Objects repelled each other and the screen borders in a physically plausible way such that the angle of incidence equaled the angle of reflection. After 11 s, the objects stopped moving and participants were instructed to click on those targets that they had selected at the beginning of the trial. The selected targets turned yellow. Again, target selection needed to be confirmed by clicking on the dot in the center of the screen. This marked the end of the trial. See Fig. 1 (1st row) for an exemplary trial sequence.

The goal for participants was to identify as many targets as possible without making mistakes. For each correctly identified target, participants earned one point. For each incorrect selection, they lost one point.

#### Joint condition

##### Joint procedure

The task and procedure was identical to the Solo condition except that participants were now told that those targets that they did not select themselves will be selected and tracked by the computer partner. In other words, participants had the chance to offload as many targets as they wished to an algorithm. Thus, at the start of each trial, participants first selected their targets and confirmed their selection, and the remaining targets were then highlighted in violet to indicate that these had been selected by the computer partner (see Fig. 1). For example, if a participant selected four out of the six targets, the remaining two targets were automatically selected by the computer partner. Then all objects started moving across the screen (with targets and distractors looking identical). After 11 s, the objects stopped moving and participants were asked to click on their previously selected targets. After participants confirmed their selection, the remaining targets were selected by the computer partner. The targets selected by the participant turned yellow; those selected by the computer turned violet. Participants then confirmed with the space bar that they had seen the computer’s selection, thereby marking the end of the trial. See Fig. 1 (2nd row) for an exemplary trial sequence.

##### Joint task goal

The goal for participants was to identify, jointly with the computer partner, as many targets as possible without making mistakes. As in the Solo condition, participants earned one point for each correctly identified target and lost one point for each incorrect selection. Importantly though, the same rule applied to the targets selected by the computer partner such that the total score consisted of the sum of participants’ own and the computer’s points.

Note that in our previous study (Wahn et al., 2023), participants also had the option to offload an unlimited number of targets to the computer in the Joint condition. We then compared how many targets participants chose to track in the Joint condition relative to the Solo condition. If, for example, a participant chose to track two targets in the Joint and three targets in the Solo condition, this meant that in the Joint condition they had “offloaded” one target to the computer. Importantly, in our previous study, participants were free to offload all targets to the computer (i.e., the entire MOT task). However, this would leave them effectively without any task to perform themselves. The results from our previous study showed that participants did not offload the entire task. As mentioned above, potential explanations for this finding are that participants did not want to feel bored while passively waiting for the computer to finish the task, or that they did not want to fall short of the experimenter’s demand (i.e., they felt that they could not simply sit back and wait for the computer to do the task but needed to contribute something).

In the present study, we aimed to exclude that these two potential factors (boredom and experimenter demand) could influence participants’ offloading decision. Therefore, we introduced a bonus task that participants could perform if they decided to completely offload the MOT task to the computer. As a bonus task, we chose an established pen and paper mental rotation task (Vandenberg & Kuse, 1978; we used the version by Peters et al., 1995). It required participants to mentally rotate a target figure in order to identify with which of four rotated figures it matches (see bottom row of Fig. 1, for an example). In fact, two of the four rotated figures matched the target figure but participants needed to identify only one. We chose this mental rotation task as a bonus task because it requires sustained spatial attention, just like the MOT task. This way, we made sure that participants performed either the MOT task or the mental rotation task—it was not possible to properly perform both spatial tasks at the same time (e.g., Wahn & König, 2015). Thus, participants had to focus their attention either on the computer screen (MOT task) or on the paper in front of them (mental rotation task).

In each trial, participants could decide whether to offload the entire MOT task (i.e., to offload all targets) or not (i.e., to offload only a subset of targets, or no targets at all). If they chose to offload the task completely, this meant that the computer partner tracked all six targets while the participant could complete one item of the mental rotation bonus task. Participants could adjust their offloading choice on a trial-by-trial basis. For example, they might choose to offload the MOT task completely in one trial and perform the bonus task, and, in the next trial, they might choose to track three targets and offload three targets to the computer.

##### Bonus task incentive in joint condition

Importantly, participants were told that they could earn additional money for each correctly solved mental rotation item (10 cents per item). Thus, if participants decided to completely offload the MOT task in all 50 trials, and if they solved all mental rotation items correctly, then they would earn 5 EUR (50 × 10 cents) in addition to the base payment of 15 EUR. However, this additional financial gain was conditional on the fact that the overall accuracy in the MOT task was at least 90%. If this accuracy threshold was not met, the bonus task would not be rewarded and 5 EUR would be deducted from participants’ base payment such that they would receive only 10 EUR in the end. The payoff structure was implemented in this way to convey to participants that they should only offload the entire MOT task if they trusted the computer, i.e., if they believed that the computer would perform the MOT task with high accuracy. If the computer performed the MOT task accurately and participants solved all bonus task items accurately, then participants’ total reward would be 20 EUR. However, if the computer did not perform the MOT task accurately, participants would end up with 10 EUR only (regardless of their own performance in the bonus task). In fact, the computer’s tracking accuracy was 100% (i.e., its target selections were always correct) but participants in Experiment 1 were not informed about this. If participants did not engage in offloading at all (i.e., did not perform the bonus task), they would receive the base payment of 15 EUR.

#### Questionnaires

After participants completed the Solo and Joint conditions, they were asked if they had completely offloaded the MOT task to the computer partner (in some or all trials). They were also asked to specify their reasons for offloading. Note that the word “offload” was not explicitly used in these questions. Furthermore, participants were asked to indicate how many targets they believed the computer partner could track accurately (on a scale from “0 targets” to “more than 6 targets”). The last option on the scale (“more than 6 targets”) was included to test whether participants believed that the capacity of the computer was virtually unlimited such that it could, in principle, also track more than six targets (if more were available).

Next, participants were asked to fill in a set of questionnaire items that capture personal characteristics. We hypothesized that participants’ replies to these items might be informative with regard to the factors that influence a person’s willingness to offload tasks to an artificial system. The set consisted of the Desirability of Control Scale (20 items; Burger & Cooper, 1979), the Trust in Automation Scale (3 items-subset; Körber, 2019), and the Affinity for Technological Systems Scale (9 items; Franke et al., 2019). The three items from the Trust in Automation Scale were asked in their original wording. In addition, we adapted the remaining seven items from that Scale such that the items now referred specifically to the computer partner from the experiment (rather than to automated systems in general). This adaption was done to understand how participants perceived the reliability and competence of the computer partner (rather than of any generic automated system). All questionnaire items (in German), as well as links to the English translation, are provided in the Additional file 1: Supplementary Material S2.

#### Experiments overview

We conducted three experiments. In each experiment, a different group of participants underwent exactly the same procedure. The computer’s tracking accuracy was 100% in all experiments. The crucial difference between Experiment 1 versus Experiments 2 and 3 was that in Experiments 2 and 3, participants were explicitly informed about the computer’s capacity (“computer capacity known”; see Additional file 1: Supplementary Material S2 for the exact wording in German) whereas participants in Experiment 1 were not informed (“computer capacity unknown”). The crucial difference between Experiment 3 versus Experiments 1 and 2 was that in Experiment 3, the bonus task came *without* any potential additional financial gain; all participants in Experiment 3 were paid the base payment of 15 EUR.

#### Technical

We programmed the experiments in *Python* 3.0 with the *pygame* library. Participants took about 75 min to complete the experiment and the questionnaires. We performed all analyses in *R* using customized *R* scripts.

### Data analysis

For all pairwise comparisons (i.e., comparisons between *two* within- or between-subjects conditions), we used dependent and independent *t*-tests. For within-subjects comparisons that involved more than two conditions and/or more than one factor, we used linear mixed models (LMMs). We chose LMMs instead of within-subjects ANOVAs because LMMs allow for a more precise modeling of the data (via the inclusion of random intercepts and slopes for each participant) and because, with LMMs, it is possible to include non-categorical factors (e.g., time course). For between-subjects comparisons that involved more than two conditions and/or more than one factor, we used a multiple linear regression (MLR). We chose a MLR instead of a between-subjects ANOVA because we aimed to perform a step-wise regression to assess the variance explained by our questionnaire scales on top of the variance explained by the experimental factors. For comparing frequencies, we used a standard Chi-squared test.

We will first report the results for Experiments 1 and 2, focusing on the knowledge manipulation (computer capacity known/unknown), see Fig. 3. Next, we will report an analysis that combines data from Experiments 1 and 2 with the two experiments from our previous study (Wahn et al., 2023). This analysis gives us a 2 × 2 between-study design with the between-study factor “Bonus task” (with, without), with a bonus task present in the current but absent in our previous study, and the within-study factor “Computer capacity” (known, unknown); see Fig. 6. Finally, we will report the results for Experiment 3, focusing on the incentive manipulation (bonus task with/without incentive). To do so, we compare Experiment 3 (bonus task without incentive) to Experiment 2 (bonus task with incentive) and to Experiment 2 from our previous study (no bonus task); see Fig. 7.

## Results

### Experiments 1 and 2: Does knowledge about the computer’s capacity affect offloading?

**Fig. 2:**
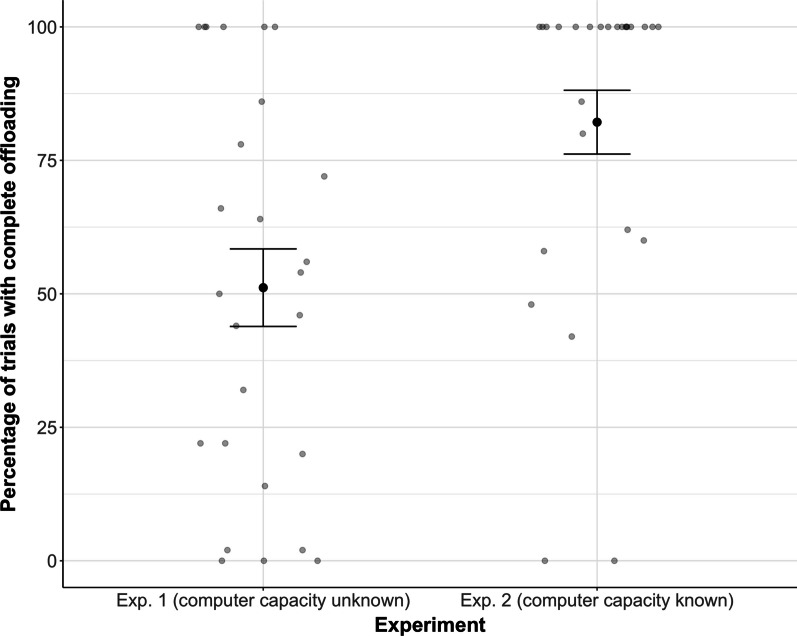
Percentage of trials in which participants completely offloaded the MOT task to the computer partner, shown separately for Experiment 1 (left) and Experiment 2 (right). Error bars are Standard Error of the Mean

To address the question of whether humans are willing to completely offload an attentionally demanding task to an algorithm, we first computed the percentage of trials in which participants offloaded all targets to the computer partner and performed the bonus task instead. We found that participants completely offloaded the MOT task in 51% of trials in Experiment 1 and in 82% of trials in Experiment 2 (see Fig. 2 for a descriptive overview). The offloading frequency was significantly higher in Experiment 2 compared to 1 (*t*(50) = 3.30, *p* = 0.002, Cohen’s *d* = 0.91), indicating that participants who received information about the computer partner’s accuracy beforehand (in Experiment 2) were more likely to offload the entire task. Note that participants’ offloading behavior did not change throughout the course of the experiment (see Additional file 1: Supplementary Material S1 for additional analysis).

**Fig. 3:**
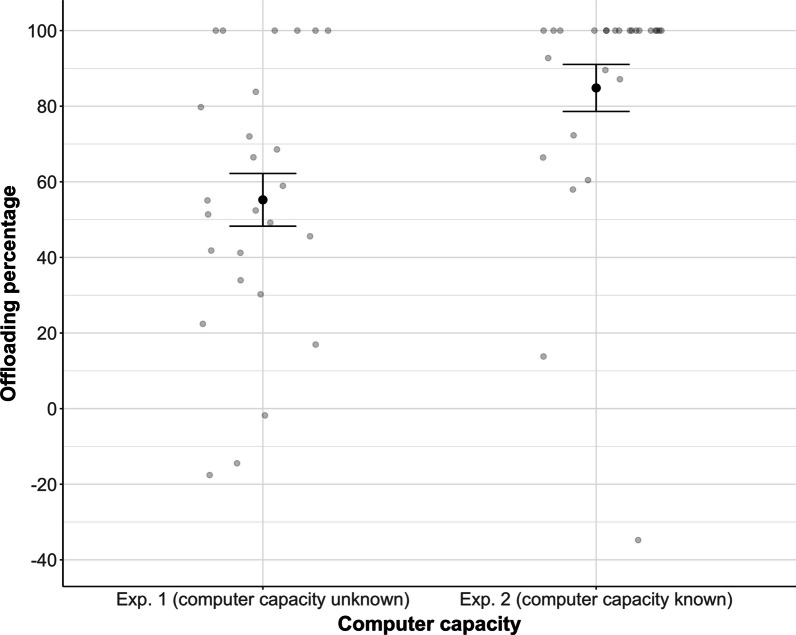
Offloading percentage for Experiment 1 (left) and Experiment 2 (right). Errors bars are Standard Error of the Mean

After having looked at the specific case of *complete* offloading (i.e., taking into account only trials in which participants offloaded the *entire* MOT task), we then analyzed participants’ overall—partial and complete—offloading behavior. For this purpose, we calculated an “offloading percentage” to capture the relative tracking load that participants offloaded to the computer partner. This percentage was computed as the number of targets participants offloaded in the Joint condition divided by the number of targets they tracked in the Solo condition, multiplied by 100. If, for instance, a participant tracked three targets in the Solo condition but only one target in the Joint condition, this participant offloaded two targets to the computer. Their offloading percentage would thus be 2/3 targets, hence 66%. If participants chose to perform the bonus task in all trials of the Joint condition, their offloading percentage would be 100%. When comparing participants’ offloading percentage between Experiments 1 and 2, we found that it was significantly higher in Experiment 2 (85%) compared to Experiment 1 (55%), *t*(50) = 3.17, *p* = 0.003, *d* = 0.88. For a descriptive overview, see Fig. 3.

In a next step, we aimed to rule out alternative explanations for why the observed offloading behavior differed between experiments. Specifically, we tested whether participants’ performance in the Solo condition (tracking accuracy and number of selected targets) differed between experiments. Theoretically, if participants differed in their individual performance in the Solo condition, this might also explain differences in the Joint condition. For example, participants in Experiment 2 might have found the MOT task *generally* more difficult (in the Solo condition) and thus might have preferred to offload it more often in the Joint condition. Comparing tracking accuracy and number of selected targets between experiments, we found that participants performed with high accuracy both in Experiment 1 (*M* = 0.95, SD = 0.05) and Experiment 2 (*M* = 0.94, SD = 0.07). Accuracy did not differ significantly between experiments (*t*(50) = 0.60, *p* = 0.549, Cohen’s *d* = 0.17). Secondly, we found that, on average, participants tracked the same number of targets in the Solo condition in Experiment 1 (*M* = 3.11, SD = 0.47) and Experiment 2 (*M* = 3.11, SD = 0.67). Again, there was no significant difference between experiments (*t*(50) = 0.01, *p* = 0.992, Cohen’s *d* = 0.00). Thus, we can rule out that the observed differences in offloading behavior between experiments can be alternatively explained by differences in individual performance. Note that both tracking accuracy and number of selected targets were also similar in the Solo condition of our previous study (Wahn et al., 2023).

Furthermore, we checked participants’ accuracy in the mental rotation task and found that participants performed with high accuracy in Experiment 1 (*M* = 0.84, SD = 0.21) and Experiment 2 (*M* = 0.89, SD = 0.09). Accuracy did not differ significantly between experiments (*t*(44) = 0.91, *p* = 0.367, Cohen’s *d* = 0.27).

**Fig. 4:**
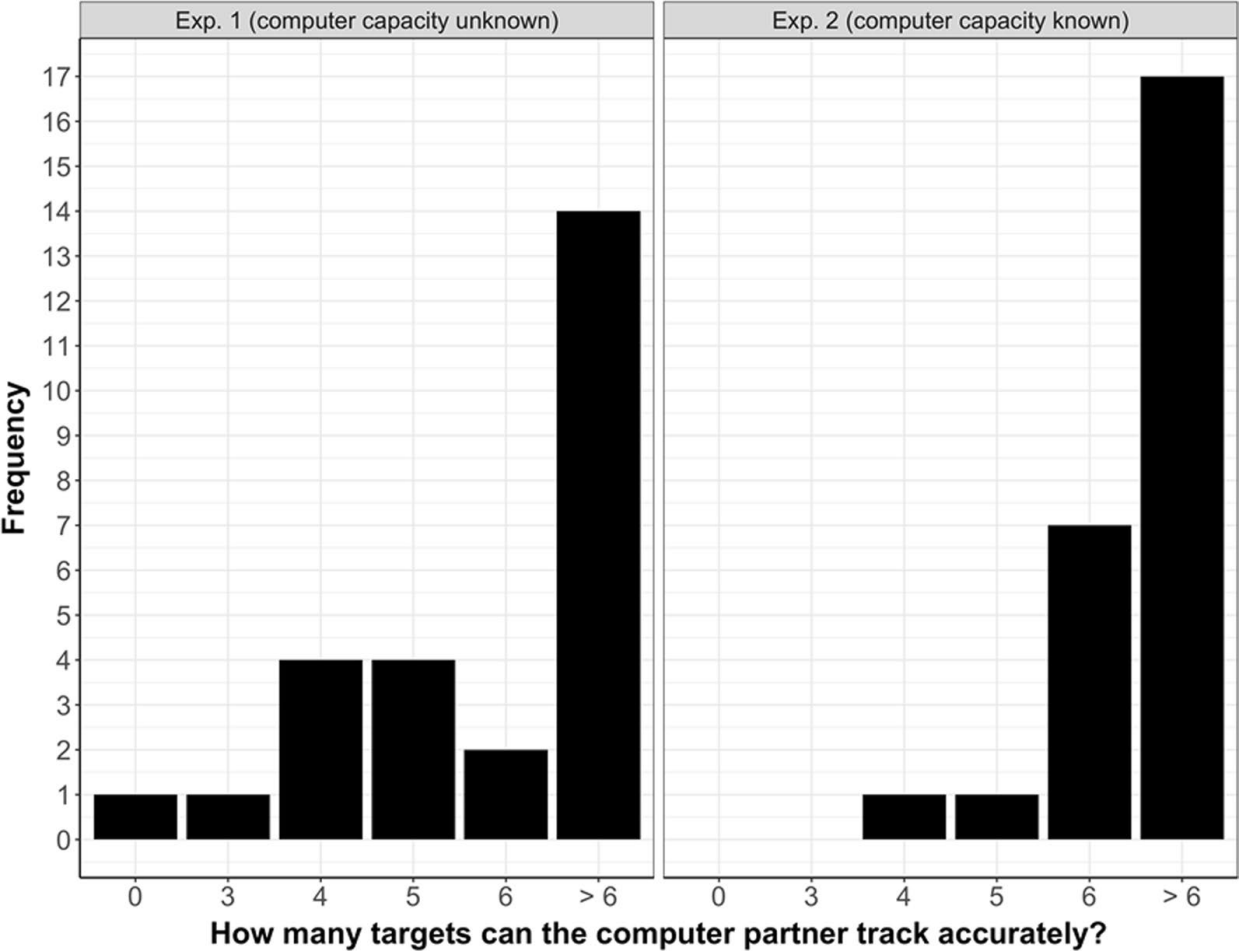
Rated capacity of the computer partner in Experiment 1 (left) and Experiment 2 (right)

However, before being able to attribute the difference in offloading behavior to participants’ prior belief about the computer’s accuracy, we needed to check that participants in Experiment 2 indeed believed the prior information we provided them with (i.e., the statement that the computer’s accuracy was 100%). Thus, we assessed how participants rated the computer’s capacity in Experiment 2 and found that the majority of participants (24/26) rated it as 6 (or more) targets, with six being the maximum number of to-be-tracked targets (see Fig. 4 for a descriptive overview). This suggests that most participants believed the prior information and consequently assumed that the computer’s tracking capacity was high enough to completely take over the MOT task. In contrast, in Experiment 1, only ~ 60% of the participants (16/26) rated the capacity as 6 (or more) targets. Comparing the frequencies between experiments using a *χ*^2^ test, we found that they did not differ significantly (*χ*^2^(5) = 8.17, *p* = 0.123).

To get an understanding of the reasons behind participants’ decision to completely offload the MOT task and perform the bonus task instead, we categorized participants’ responses to the open-ended question (“What were your reasons for completely offloading the MOT task to the computer partner?”) into the following four categories:

1. *Avoid boredom* Participants wanted to perform the bonus task to stay engaged, not feel bored, to have fun, or to meet a new challenge.
2. *Monetary gain/productivity* Participants wanted to perform the bonus task to earn more money in total. Two participants said that they aimed to increase the overall productivity (i.e., their own and the computer’s productivity combined); we classified these responses into the same category.
3. *Trust the computer* Participants trusted the computer partner to perform the MOT task accurately. Participants either said that they trusted the computer blindly or they specified that they first checked the computer’s accuracy by monitoring the computer’s performance in the MOT task for a few trials.
4. *No offloading* Participants did not completely offload the MOT task to the computer partner.

**Fig. 5:**
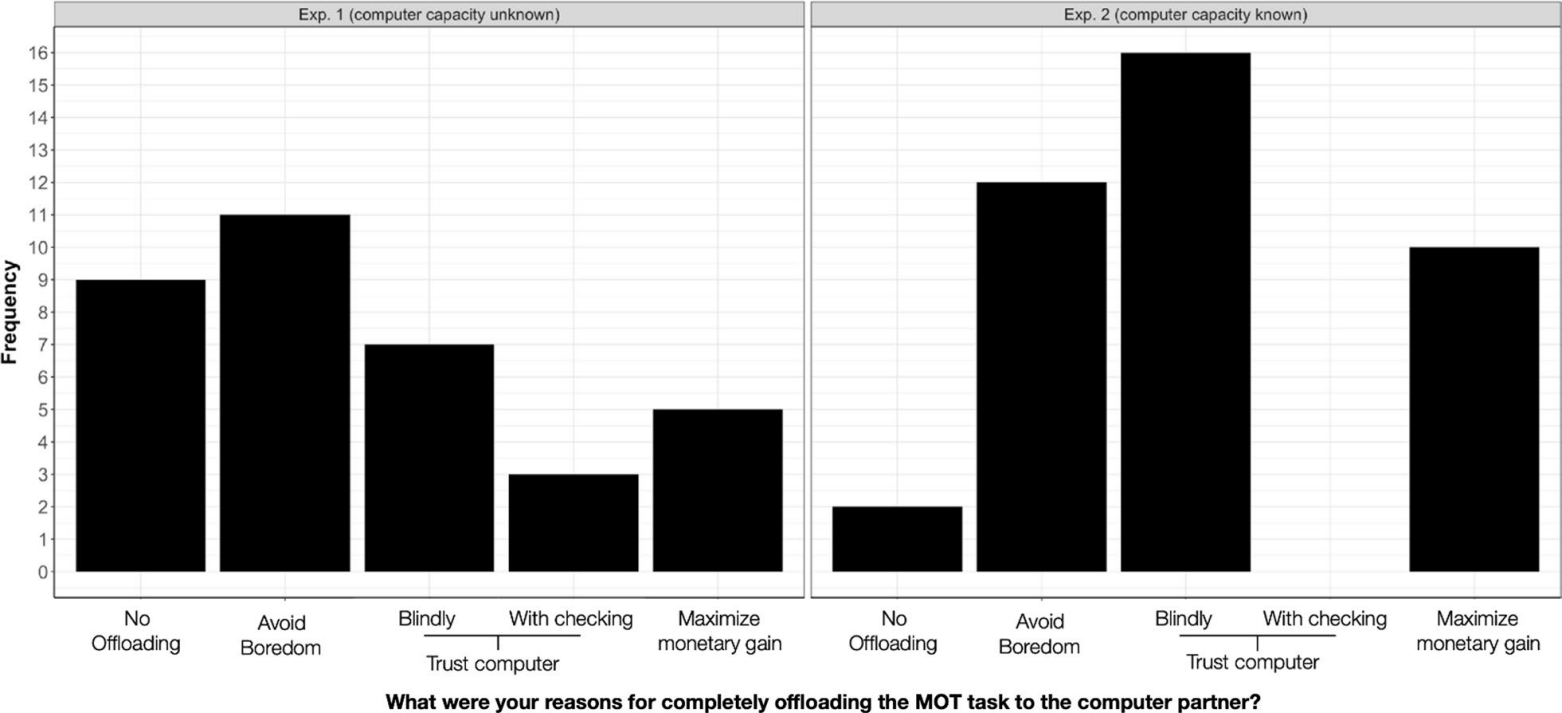
Reasons for offloading in Experiment 1 (left) and Experiment 2 (right)

Figure 5 shows an overview of the frequencies with which these four responses were reported. Note that participants could also provide multiple responses—indeed, eight participants in Experiment 1 and 13 participants in Experiment 2 provided more than one reason for offloading. We included all responses into the frequency distribution. On a descriptive level, we found that “Avoid boredom” has a high frequency in both experiments and that “Trust computer” has a considerably higher frequency in Experiment 2 (16x) compared to Experiment 1 (10x). When comparing experiments using a Chi Square test, we found that the overall frequency distribution significantly differed (*χ*^2^(4) = 12.41, *p* = 0.015). Note that when we excluded all “No offloading”-responses, the comparison was not significant (*χ*^2^(3) = 6.20, *p* = 0.102).

### Experiments 1 and 2 versus previous study: Does a bonus task facilitate offloading?

**Fig. 6:**
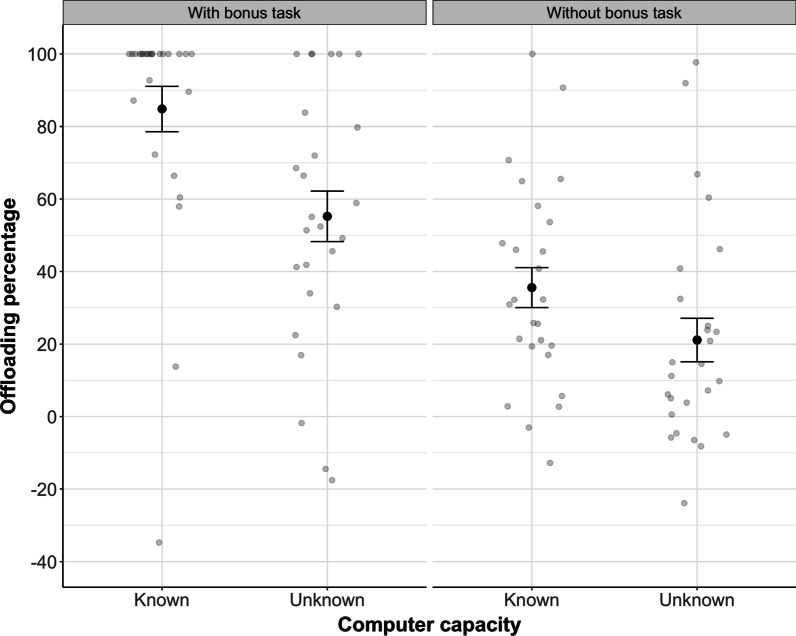
Offloading percentage, displayed as a function of Bonus task (with, without) and Computer capacity (known, unknown). Note that the data in the left panel (“with bonus task”) represent Experiment 1 (Unknown) and 2 (Known) from the present study (compare Fig. 3), whereas data in the right panel (“without bonus task”) are taken from Experiments 1 and 2 from our own previous study (Wahn et al., 2023). Errors bars are Standard Error of the Mean

We next assessed how the offloading extent differed between the present and our previous study (Wahn et al., 2023). To be able to compare experiments in the two studies, we introduced the between-study factor “Bonus task” (with, without), with a bonus task present in the current but absent in our previous study (Wahn et al., 2023). The second, within-study factor was “Computer capacity” (known, unknown), which captures the difference between Experiment 1 (in which participants were not informed about the computer’s capacity) and Experiment 2 (in which participants were informed that the computer’s tracking accuracy was 100%). This factor was the same in our present and previous study. For a descriptive overview of the results, see Fig. 6.

We computed a MLR model with offloading percentage as dependent variable and the categorical factors Bonus task (with, without) and Computer capacity (known, unknown) and the interaction term between these two factors. Overall, the model was significant (*F*(3,100) = 19.84, *p* < 0.001). Specifically, the two factors Bonus task and Computer capacity were significant but the interaction was not (see Model 1 in Table 1).^2^ These findings suggest that the availability of a bonus task as well as explicit knowledge about the computer’s capacity significantly increased participants’ tendency for offloading. A comparison of the regression weights (see Model 1 in Table 1) shows that the factor bonus task contributes more toward explaining the variance in offloading percentage than the factor computer capacity. The squared semi-partial correlations (bonus task: *sr*^*2*^ = 0.28; computer capacity: *sr*^*2*^ = 0.08), which quantify the unique variance explained by the respective factor, show that the factor Bonus task explains 28% of the variance whereas the factor Computer capacity explains 8% of the variance.

**Table 1:** Results of a linear regression model with the between-study factor Bonus task and the within-study factor Computer capacity (Model 1), and a model that additionally includes our questionnaire data (Model 2)

	Model 1	Model 2
Computer capacity	− 0.38** (8.76)	− 0.28* (8.83)
Bonus task	− 0.63*** (8.76)	− 0.58*** (8.59)
Computer capacity * Bonus task	0.17 (12.38)	0.11 (12.10)
Desire to control		− 0.03 (0.26)
Reliability/competence of computer partner		0.25** (7.13)
Affinity for technological system		0.07 (2.07)
*R*^2^	0.37	0.44
Adj. *R*^2^	0.35	0.41
Num. obs	104	104

All regression weights are standardized. As such, an increase in one standard deviation unit in the respective predictor corresponds to an increase of the beta coefficient value in the dependent variable. Standard Error of the Mean of non-standardized coefficients are in brackets

***p < 0.001; **p < 0.01; *p < 0.05

We then pooled our questionnaire data on personal characteristics from the present and previous study (Wahn et al., 2023), resulting in an *N* = 104. We assessed the reliability of all questionnaire scales using Cronbach’s alpha (Cronbach, 1951) and found that the scales Desirability of Control (*r* = 0.80), Affinity for Technological Systems (*r* = 0.90) and Reliability and Competence of the Computer Partner (*r* = 0.79) had satisfactory reliabilities. The reliability of the Trust in Automation scale, however, was too low (*r* = 0.60), and the scale was thus not included in the analysis (a Cronbach’s alpha of at least 0.70 is considered acceptable for research purposes; see Taber, 2018). In a step-wise regression, we added the three reliable questionnaire scales as continuous predictors to our MLR model above. A model comparison showed that the addition was significant (*F*(3,97) = 4.13, *p* = 0.008), suggesting that the questionnaire scales explain 7% additional variance. When examining the model predictors, we found that Reliability and Competence of the Computer Partner was a significant predictor whereas the other two predictors were not (see Model 2 in Table 1). This correlational finding, however, must be interpreted with a bit of caution given that our current sample size (*N* = 104) may not suffice to obtain a stable correlation (see Schönbrodt & Perugini, 2013).

### Experiment 3: What role does a financial incentive play?

Combining the results from Experiments 1 and 2 with the results from our previous study showed that the availability of a bonus task facilitated participants’ willingness to offload such that participants showed an increase in offloading when a bonus task was available (present study) compared to when it was not (previous study). However, it is an open question whether participants in the present study chose to perform the bonus task simply because they wanted to stay engaged or because they aimed to gain additional money. The questionnaire results (see Fig. 5) show that some participants wanted to avoid boredom but others wanted to maximize monetary gain. To disentangle the influence of these two factors on offloading behavior, we carried out Experiment 3. As mentioned above, Experiment 3 was identical to Experiment 2 with the only difference that the bonus task came without financial incentive, i.e., participants could neither earn nor lose any money (all received the same base payment).

In Experiment 3, participants completely offloaded the MOT task in 57% of all trials (Exp. 1: 51%, Exp. 2: 82%). The overall offloading percentage was 68% (Exp. 1: 55%, Exp. 2: 85%). Participants’ accuracy in the mental rotation task was high (*M* = 0.89, SD = 0.11), comparable to Experiments 1 (*M* = 0.84, SD = 0.21) and 2 (*M* = 0.89, SD = 0.09). As in Experiment 2, the majority of participants (25 out of 26) rated the computer’s capacity as 6 (or more) targets, suggesting that participants in fact believed that the computer was able to perform the MOT task accurately. When asked why they offloaded the task to the computer, 11 participants reported that they wanted to avoid boredom and 10 participants said that they trusted the computer.

**Fig. 7:**
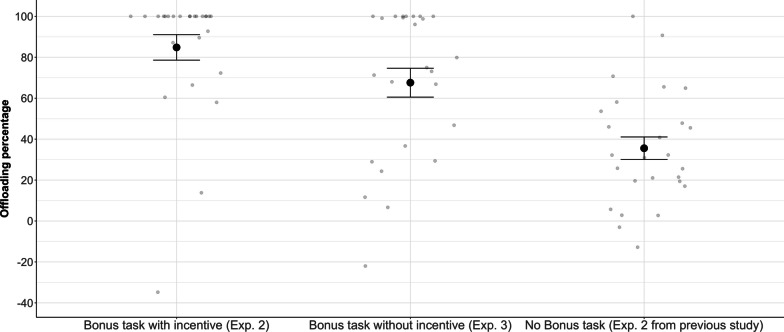
Offloading percentage as a function of monetary incentive and bonus task availability. Errors bars are Standard Error of the Mean

To focus on the effect of the availability of an (incentivized) bonus task, we chose to compare the offloading percentage across three experiments (see Fig. 7): Experiment 2 from our previous study (no bonus task), Experiment 2 from the present study (bonus task with incentive), and Experiment 3 from the present study (bonus task without incentive). In all of these selected experiments, participants were explicitly informed about the computer partner’s capacity.

We ran a MLR model with “Experiment” as between-subject factor and “offloading percentage” as dependent variable, specifying “Experiment 3” as a reference group. This model tests if there is a main effect of Experiment and it also runs pairwise comparisons between Experiment 3 and the other two experiments. Results showed that the model was significant, *F*(2,75) = 15.75, *p* < 0.001, *R*^2^ = 0.30. The offloading percentage was significantly higher (by 32%) for Experiment 3 relative to Experiment 2 from the previous study without any bonus task (*t* = − 3.60, *p* = 0.001), suggesting that the addition of a bonus task increased participants’ willingness to offload. The offloading percentage between Experiment 3 (without incentive) and Experiment 2 (with incentive) also differed numerically (68% vs. 85%), yet this difference was not significant, *t* = 1.93, *p* = 0.057.

## Discussion

In the present study, we investigated under which conditions people are willing to offload an attentionally demanding task (i.e., a multiple object tracking (MOT) task) to an algorithm. We assessed whether people’s willingness to offload is affected by the availability of an (incentivized) bonus task and by participants’ knowledge about the algorithm’s capacity. Additionally, we assessed whether certain personal characteristics predict offloading and we also aimed to find out about people’s subjective reasons for offloading.

We based this study on the results of a recent study of our own (Wahn et al., 2023), with the goal to extend those results and clarify open questions. In our previous study, participants performed the same MOT task as in the present study. This task requires participants to track a subset of moving targets among distractors on a computer screen. Participants in our previous study first performed the MOT task alone and then had the opportunity to offload an unlimited number of targets to a computer partner (which was effectively an algorithm). Participants chose to offload the MOT task partially, but never completely, to the computer partner; i.e., they always continued tracking a subset of targets themselves. We hypothesized that participants’ offloading behavior might have been influenced by their wish to avoid boredom and/or to meet the experimenter’s demands. That is, their decision not to offload the complete task but to continue performing (at least parts of) the task themselves might not have been motivated by potential distrust toward the computer partner but rather by the wish to stay engaged (instead of passively waiting for the computer to finish the task) and/or by the experienced duty to complete the “work” they were being paid for in the context of the experiment.

The aim of the present study was to understand whether the aforementioned reasons might have prevented participants in our previous study from offloading the entire task to the computer partner. To this end, we adjusted the task in a way that should facilitate participants’ willingness to offload. Specifically, we kept everything comparable to our previous study except that now we informed participants that, if they were to offload the MOT task completely to the computer partner, then they would be able to perform a bonus task while the computer performed the MOT task. This way, participants should feel free to offload the entire task—they should not feel bored nor should they have the impression to fall short of the experimenter’s demands. Successful performance in the bonus task resulted in additional financial gain (in Experiments 1 and 2). However, this gain was conditional on a high performance accuracy in the MOT task. Thus, participants should only offload the MOT task if they trusted the computer to perform this task accurately. In a third Experiment, we tested to what extent the additional financial gain actually affected participants’ offloading willingness.

It is worthwhile mentioning that in most previous research, the term offloading is understood as *partial* offloading, in the sense that participants could offload only parts of a task—and not the entire task—while performing the remaining parts themselves. In contrast, in the present study, we were also interested in whether and under which conditions people are willing to offload an entire task. Yet, of course there is a clear difference between delegating only parts of a task (to reduce cognitive demand, see Risko and Gilbert (2016)) and handing over an entire task (to free resources for another task). We thus suggest to introduce the distinction between what we have here called *partial* and *complete* offloading (i.e., in the present task, the distinction between offloading a subset of to-be-tracked targets vs. all targets).

As in our previous study and in line with earlier research (Weis & Wiese, 2019a, 2019b, 2022; Wiese et al., 2022), we included “knowledge of the computer’s capacity” as an additional factor in the present study. That is, we varied whether participants were informed about the computer’s capacity (Experiment 2 and 3) or not (Experiment 1). As in our previous study, the computer’s tracking accuracy was 100% in all experiments, i.e., it always performed the MOT task flawlessly regardless of how many targets it was assigned to track.

We found that participants completely offloaded the MOT task on average in ~ 50% of all trials (Experiment 1), i.e., they performed an equal amount of trials in which they offloaded the complete MOT task to the computer while performing the bonus task themselves, and trials in which they offloaded the MOT task only partially and continued tracking a subset of targets themselves. The willingness to completely offload increased significantly (up to 80%) if participants were informed beforehand that the computer’s tracking accuracy was flawless (Experiment 2). To test whether the availability of a bonus task affected participants’ willingness to offload, we compared the results of Experiments 1 and 2 of the present study with the results from our previous study which did not involve a bonus task but was identical otherwise. When comparing the offloading percentage (i.e., the percentage of the task load that participants offloaded to the computer partner relative to the Solo condition) across studies, we found that participants’ offloading percentage was significantly higher in the present study compared to our previous study (Wahn et al., 2023; 70% vs. 28%). This suggests that the availability of a bonus task facilitates people’s willingness for offloading. Together, our findings indicate that people's willingness to offload an attentionally demanding task to an algorithm is boosted by the knowledge about the algorithm’s capacity and by the availability of a bonus task (see Fig. 6).

When asked about their reasons for completely offloading the task to the computer, participants in the present study most frequently reported that they offloaded the task because they trusted the computer and because they wanted to try out the bonus task to avoid boredom and stay engaged. Thus, on the one hand, people wanted to make sure that the offloaded work was performed well: they were more willing to offload if they had information about the computer’s reliability and could thus trust it blindly (see Experiment 1 vs. 2). Moreover, it mattered that people did not face the alternative of doing nothing when offloading their work: they were more willing to offload if they themselves had another task to perform (present study with bonus task vs. previous study without bonus task). This latter finding is in line with recent research showing that people sometimes rather choose to engage in cognitive effort if the alternative is doing nothing (Wu et al., 2023). Indeed, our study suggests that the primary motivation for people’s willingness to offload a task may not always be the reduction of cognitive effort but rather, for instance, the motivation to avoid boredom and try out something new.

In addition to the two reasons mentioned above, participants also reported that they performed the bonus task to maximize monetary gain—consistent with the theoretical account that people choose offloading insofar as it maximizes reward (Gilbert, 2023). To address this factor, we carried out Experiment 3 in which participants had the option to perform a bonus task (just as in Experiment 2) but did not receive any additional financial reward for it. However, we did not find a significant difference between the offloading behavior in Experiment 3 (without incentive) and Experiment 2 (with incentive). Taking into account also the results from our previous study, we conclude that the mere availability of a bonus task facilitates people’s willingness for offloading, even if no additional financial incentive is provided, see Fig. 7.

In fact, in everyday life, it seems that such explicit financial incentives are rare and that people instead benefit more indirectly from complete offloading. Consider, for example, offloading a certain task (e.g., writing a business email) to *ChatGPT*. This will save you time and cognitive resources to perform another (maybe more interesting?) task yourself. By doing so, you might benefit in various ways: you might be happy to avoid writing a boring email, you might enjoy the alternative task more, and you might also benefit in terms of efficiency and finish your work’s task load earlier than expected.

When analyzing to what extent our questionnaire scales explain additional variance on top of the two factors we manipulated in our experiments (i.e., knowledge about the computer partner’s capacity and bonus task availability), we found that the rated competence of the computer partner is the only significant predictor. Our other questionnaire scales—Desirability of Control and Affinity for Technological Systems—did not explain additional variance. This suggests that when controlling for the two before-mentioned factors, variance in participants’ offloading behavior can be additionally explained by participants’ explicit ratings of the computer’s competence. This finding further corroborates the idea that the perceived competence of an artificial system plays an important role in the context of offloading, as not only our experimental manipulation (computer capacity known vs. unknown) leads to significant differences in offloading but also the rated competence of the computer positively correlates with offloading. These findings are in line with and extend earlier work which showed that the perceived competence of an artificial system influences the extent to which people are willing to share a task with it (Weis & Wiese, 2022). Note though that the present correlational finding should be interpreted with a bit of caution given that our sample size (*N* = 104) may not suffice to obtain a stable correlation (see Schönbrodt & Perugini, 2013).

## Limitations

We note at this point that the above conclusions partially rest on a comparison between data from the present study and data from a previous study of ours (Wahn et al., 2023). Even though such comparisons across studies are not standard practice, we believe that in the present case, this type of between-study comparison can be justified. First of all, we suggest that in any case, the factor bonus task (with, without) must be manipulated in a between-subject design. This is because a within-subject design would likely lead to order/carry-over effects and would considerably lengthen the experiment which, in turn, could negatively affect participants’ attentional performance due to fatigue effects. We thus chose to conduct a between-subject study (by including data from our previous experiments) while ensuring the highest possible standards of comparability between groups (see p. 10). To this end, we made sure that participant samples did not differ in terms of demographics and that the experimental conditions (location, equipment, experimenter) are exactly the same in the present and previous study.

Another limitation of the present study is the sample size, which was matched to our previous study but was originally computed for correlations and pairwise comparisons, yet not for more complex analyses (i.e., LMMs or MLRs). A replication study with an adjusted sample size would consolidate the current findings.

## Future directions

Future research could further investigate people’s “desire to stay engaged/avoid boredom” as a reason for offloading by varying the engagement level of the task that people can potentially offload. It is possible that people might be more willing to offload a boring task to an algorithm compared to an engaging task (even if the competence of the algorithm is unknown). Instead (or in addition to) the engagement factor of the task, one could also vary the task type in order to shed light onto people’s preconceptions about the capacities of artificial systems. This could extend earlier work which showed that people were more willing to offload an arithmetic task to an algorithm compared to an emotion judgment task (Wiese et al., 2022).

One further possible motivation for why participants in the present study chose to offload the task completely (rather than partially) might have been that participants aimed to minimize switch costs which would occur when changing back and forth between the MOT task on one trial and the mental rotation task on the next trial. While none of our participants explicitly mentioned this motivation, it may still be worth exploring this further.

Relatedly, a future study could explore offloading in a dual-task design where two attentionally demanding tasks need to be performed at the same time (for a recent review see Wahn & Sinnett, 2019). Investigating this could be worthwhile because, in attentionally demanding professions (e.g., in aviation, air-traffic control, and car-driving), workers are often faced with dual-task demands and offloading parts of these demands could help prevent costly errors. A recent study (Grinschgl et al., 2023) used a dual-task design with two memory tasks (i.e., a visual pattern copy task and an auditory N-back task). If participants offloaded parts of the copy task, their performance in the N-back task increased. This suggests that in dual-task scenarios in the domain of memory, offloading cognition in one task can benefit the simultaneous performance in the second task. An open question is whether this finding translates to the domain of attention. To address this question, a future study could test to what extent humans are willing to offload tracking load in a MOT task if they are required to simultaneously perform an auditory localization task (Wahn & König, 2015).

Moreover, future research could tap more directly into the limitations of attention (Marois & Ivanoff, 2005; Wahn & König, 2017) by instructing participants to track a number of targets that goes beyond their individual maximum tracking load and thereby create an attentional overload. With this manipulation, participants might be willing to offload to a larger extent (compared to the present study) as they would likely feel overwhelmed with the task in the Solo condition. In other words, the experienced attentional overload may create an underestimation of one’s own tracking abilities, which in turn could lead to more offloading. Another direction for future research could be to introduce errors in the computer partner’s performance. In this case, if participants initially monitor the computer’s performance, they might stop offloading after witnessing the computer’s errors. This is predicted based on earlier research which showed that humans tend to lose trust in an algorithm once it commits an error (Dietvorst et al., 2015). Alternatively, participants might not monitor the computer’s performance at all and simply trust that it will perform accurately—they would thus not even notice the computer’s errors. This is predicted by previous research on the so-called automation bias, which has been defined as “the use of automation as a heuristic replacement for vigilant information seeking and processing” (Mosier & Skitka, 1999, p. 344). Thus, a future study could test these two alternative predictions and determine whether errors in the algorithm’s performance lead to a drop in offloading because people lose trust in the algorithm or whether errors actually do not affect offloading because people’s automation bias prevails.

## Conclusions

In sum, the present study extends earlier work on cognitive offloading (Wahn et al., 2023; Weis & Wiese, 2019a, 2019b, 2022; Wiese et al., 2022) by showing that people's willingness to offload an attentionally demanding task to an algorithm is critically boosted both by the availability of a bonus task and by the knowledge about the algorithm’s capacity. This finding has potential applied relevance in today’s world in which, on the one hand, people often perform more than one task at a time, increasing their attentional load and thus the risk for errors (e.g., Wahn & König, 2015), and, on the other hand, cognitive tasks formerly reserved for humans have become feasible for artificial systems. Hence, people could substantially profit from offloading one task (of several) to an artificial system, thereby decreasing their attentional load and thus the risk for errors. The present findings suggest that people might be more inclined to offload tasks if they know that the system is capable of performing the assigned task and if they have the option to perform another task themselves.

### Supplementary Information

**Additional file 1 (containing both S1 and S2)**. **S1**: Additional analysis Exp. 1 and 2: Development of complete offloading across time. **S2**: Prior instruction for Exp. 2 and 3 as well as Questionnaires.

## Data Availability

All data for Experiments 1–3 and the analysis script are available in the following repository: https://osf.io/twh35/?view_only=8ff6f0eb23114ce78be6c8f0b4710663. The data from our previous study (Wahn et al., 2023) is available here: https://osf.io/qbgm3/?view_only=d5cd63e768d149e48d99e68292ea2609

## Footnotes

1 Please note that the participant sample from our previous study (Wahn et al., 2023) was also comparable to the present study in terms of demographics. Moreover, both studies were conducted by the same experimenter at the same university in the same experimental laboratory. Finally, all five experiments from the previous and present study combined took place within the time span of approximately 18 months. There was no participant overlap between any of the experiments.

2 Please note that the same result pattern is obtained with a standard ANOVA.
